# Modification of velopharyngeal closure pressures during phonation by neuromuscular electrical stimulation in healthy individuals

**DOI:** 10.3205/000329

**Published:** 2024-03-01

**Authors:** Simone Miller, Katharina Peters, Martin Ptok, Michael Jungheim

**Affiliations:** 1 Department of Phoniatrics and Pediatric Audiology of the Department of Otolaryngology, Hannover Medical School, Hanover, Germany; 2 Institute of General Practice and Palliative Care, Hannover Medical School, Hanover, Germany; 3 Department of Phoniatrics and Pediatric Audiology, Hannover Medical School, Hanover, Germany; 4 HNO Phoniatrie Praxis, Bremen, Germany

**Keywords:** velopharyngeal insufficiency, high resolution manometry (HRM), articulation, velopharyngeal function, neuromuscular electrical stimulation (NMES)

## Abstract

**Introduction:**

Rhinophonia aperta may result from velopharyngeal insufficiency. Neuromuscular electrical stimulation (NMES) has been discussed in the context of muscle strengthening. The aim of this study was to evaluate in healthy subjects whether NMES can change the velopharyngeal closure pattern during phonation and increase muscle strength.

**Method:**

Eleven healthy adult volunteers (21–57 years) were included. Pressure profiles were measured by high resolution manometry (HRM): isolated sustained articulation of /a/ over 5 s (protocol 1), isolated NMES applied to soft palate above motor threshold (protocol 2) and combined articulation with NMES (protocol 3). Mean activation pressures (MeanAct), maximum pressures (Max), Area under curve (AUC) and type of velum reactions were compared. A statistical comparison of mean values of protocol 1 versus protocol 3 was carried out using the Wilcoxon signed rank test. Ordinally scaled parameters were analyzed by cross table.

**Results:**

MeanAct values measured: 17.15±20.69 mmHg (protocol 1), 34.59±25.75 mmHg (protocol 3) on average, Max: 37.86±49.17 mmHg (protocol 1), 87.24±59.53 mmHg (protocol 3) and AUC: 17.06±20.70 mmHg.s (protocol 1), 33.76±23.81 mmHg.s (protocol 3). Protocol 2 produced velum reactions on 32 occasions. These presented with MeanAct values of 13.58±12.40 mmHg, Max values of 56.14±53.14 mmHg and AUC values of 13.84±12.78 mmHg.s on average. Statistical analysis comparing protocol 1 and 3 showed more positive ranks for MeanAct, Max and AUC. This difference reached statistical significance (p=0.026) for maximum pressure values.

**Conclusions:**

NMES in combination with articulation results in a change of the velopharyngeal closure pattern with a pressure increase of around 200% in healthy individuals. This might be of therapeutic benefit for patients with velopharyngeal insufficiency.

## Zusammenfassung

**Hintergrund:**

Offene Rhinophonien können aufgrund einer velopharyngealen Insuffizienz entstehen. Die neuromuskuläre Elektrostimulation (NMES) wird seit einiger Zeit als Therapieform zur Muskelkräftigung angewendet. Ziel dieser Studie war es, an gesunden Proband_innen zu untersuchen, ob NMES das velopharygeale Verschlussmuster während der Phonation verändern und die Muskelkraft erhöhen kann.

**Methodik:**

In die Studie wurden 11 gesunde Proband_innen eingeschlossen (21–57 Jahre). Mit der Hochauflösungsmanometrie wurden Druckprofile unter verschiedenen Bedingungen aufgezeichnet: die isolierte Phonation des Lautes /a/ über 5 s (Protokoll 1), die isolierte, motorisch überschwellig applizierte NMES auf das Velum (Protokoll 2) sowie die Phonation und die NMES in Kombination (Protokoll 3). Verglichen wurden der durchschnittliche Druck während der Aktivierung (MeanAct), die durchschnittlichen Maximaldrücke (Max), die durchschnittlichen Integrale unter der Druckkurve (Area under curve (AUC)) und die Art der Velumreaktion. Der Vergleich der Durchschnittswerte von Protokoll 1 und 3 wurden mithilfe des Wilcoxon-Vorzeichen-Rang-Tests durchgeführt. Ordinär skalierte Parameter wurden über eine Kreuztabelle analysiert.

**Ergebnisse:**

MeanAct Mittelwerte lagen bei 17,15±20,69 mmHg (Protokoll 1), 34,59±25,75 mmHg (Protokoll 3), Max bei 37,86±49,17 mmHg (Protokoll 1), 87,24±59,53 mmHg (Protokoll 3) und AUC bei 17,06±20,70 mmHg.s (Protokoll 1), 33.76±23.81 mmHg.s (Protokoll 3). Im Protokoll 2 zeigten sich 32 Velumreaktionen mit durchschnittlichen MeanAct-Werten von 13,58±12,40 mmHg, Maximalwerten von 56,14±53,14 mmHg und Mean-AUC-Werten von 13,84±12,78 mmHg.s. Der Vergleich der Protokolle 1 und 3 ergab mehr positive Ränge für die Kombination aus Phonation und NMES und für die Parameter MeanAct, Max und AUC. Dieser Unterschied zeigte mit p=0.026 statistische Signifikanz in Bezug auf den Parameter Max.

**Diskussion:**

NMES in Kombination mit Phonation führt bei Gesunden zu einer Veränderung des velopharyngealen Verschlussmusters mit einer Druckerhöhung von etwa 200%. Dies könnte für Patient_innen mit velopharyngealer Insuffizienz von therapeutischem Nutzen sein.

## Introduction

During articulation the velopharynx opens and closes frequently. When closed it separates the nasopharynx from the oropharynx and therewith from the nasal resonance system [1]. Different patterns of velopharyngeal closure (VPC) also exist during speech production: coronal, circular, circular with Passavant’s ridge [2], [3]. A proper closure of the velopharynx is necessary for the physiologic formation of speech sounds. Generally two movements are distinguished during the closure [3], [4]: Firstly, the soft palate elevates towards the posterior pharyngeal wall, and secondly, the lateral pharyngeal walls move medially and complete the closure. Both movements happen simultaneously and form a sphincter-like closure [3], [5]. During speech sound production the component of velum elevation is reported to be the main (but not the only) movement, whereas the lateral pharyngeal walls are reported to be less involved [6]. Closure force of the VPC was found to vary depending on sex, different sounds, and the phonetic context [6], [7].

An impaired velar function may result in an insufficient VPC, also presenting with lower pressures during the closure of the sphincter. As a result, nasal resonance may occur also for sounds that are not supposed to include nasal resonance (rhinophonia). Even after reconstructive surgery adults with a surgically repaired cleft palate (CP), for example, are reported to often continue to experience velopharyngeal insufficiency [8], [9], [10]. Lately, high resolution manometry (HRM) has been used to record pressures in the velopharynx. Whereas the velopharynx does not present with pressures during its state of rest, pressure profiles are recordable during activation [11], such as swallowing [4], [12], [13] and phonation [14]. HRM has been shown to be able to verify a functioning of the velopharyngeal closure.

In addition to surgical measures, speech and swallowing therapy [15], aiming to improve velopharyngeal insufficiencies, neuromuscular electrostimulation therapy, which has already been used for muscle strengthening in skeletal muscles [16], [17], could also be considered. Its effectiveness has also been investigated for the muscles of the pharynx in order to enhance swallowing safety of patients with dysphagia [17], [18], [19]. The application of electrical stimulation to the velum in order to improve the velopharyngeal closure has not been evaluated yet. This study therefore aims to investigate in healthy individuals whether neuromuscular electrical stimulation (NMES) does influence the velopharyngeal closure pattern during phonation. This way it shall be investigated if NMES is a potential therapeutic measure to improve velopharynx (VP) closure also for subjects with VP insufficiency.

## Materials and methods

### Study design

A monocentric prospective experimental study was conducted.

### Volunteers and patients

Eleven healthy adult volunteers (age 21–57 years, 5 male and 6 female) were included in this study. Volunteers were recruited from the medical campus of the university. Study recruitment aimed for a balanced sex and age ratio. Exclusion criteria were poor health, acute infections, pregnancy, pace makers, cancer, dysphagia, former surgery in the head, neck or throat area (in order to ensure physiology in the velopharyngeal area), chronic disease (such as arthritis, arthrosis, muscular-skeletal diseases etc.), metal implants, allergies towards metals, plastics or tooth paste. Subjects over the age of 65 were excluded to account for subclinical changes in the velopharyngeal function that might occur with higher age. All participants signed an informed consent form before undergoing any study-related procedures and were not financially remunerated. The study was approved by the institution’s ethics committee (#8611_BO_S_2019).

### Phonation tasks

A differentiated speech and voice evaluation (e.g. auditory evaluation of spontaneous speech, A-I test by Gutzmann [3]) was performed by two experienced speech and language pathologists to ensure the absence of rhinophonia.

1. All participants phonated 10 repetitions of the sound /a/ over a period of 5 seconds (protocol 1) at a regular conversational volume of between 60–70 dB.
2. For protocol 2 isolated electrostimulation was applied to the velum above the individual motor threshold or at maximum tolerable intensity (which ever one was reached first) for 5 s.
3. Protocol 3 represents the combined articulation and application of NMES either above motor threshold or at maximum tolerable intensity.

### High-resolution manometry

While the participants sat upright with the head in a neutral position, the manometric catheter was placed transnasally into the upper esophagus and fixed in place at the tip of the nose. To avoid a loss of mucosal sensitivity, a lubricating gel containing a local anesthetic agent was not used. The small-diameter catheter passed easily through the nose and was positioned to ensure that the high-pressure area of the upper esophageal sphincter (UES) and the pharyngeal structures above were represented (as described previously [4], [20], [21], [22]). Data was collected using a solid-state HRM hardware system (Solar GI HRM, Medical Measurement Systems (MMS), Enschede, The Netherlands) with a manometric catheter (Unisensor, Attikon, Switzerland) specifically designed to measure the pharynx and UES. The catheter had an outer diameter of 2 mm and a total of 20 unidirectional pressure sensors, of which 19 were spaced at 7.5 mm intervals; and one sensor was located 5 cm distal to the sensors. The catheter was calibrated and sterilized according to the manufacturer’s specifications before each measurement. All pressures were referenced to atmospheric pressure, and data was acquired at a frequency of 50 Hz for each sensor. The collected data was analyzed using MMS software (Version 8.20e).

Each participant rested for around 10 min in order to become accustomed to the catheter before performing the experimental tasks. The volume was measured using a sound level meter (Voltcraft SL-50, Hirschau, Germany).

Pressure parameters of participants were evaluated in the velopharyngeal area. Since the velopharynx (VP) stretches over an area of around 2 cm, the sensor which showed the highest pressure during activation was selected for the analysis. The parameters were provided due to the calculation function of the MMS analysis software.

1. Mean activation pressure (MeanAct): By defining the time frame of velopharyngeal activation, an average pressure value over time of the selected sensor is calculated.
2. Maximum pressure during activation: The maximum pressure (Max) value of the selected sensor is selected within the time frame of velopharyngeal activation.
3. Area under curve (AUC): This integral is calculated for the pressure curve for the selected time frame of velopharyngeal activation (mmHg.s). In order to insure comparability of the values, the AUC value was divided by the individual time of articulation.
4. The type of velum activity was classified as: no reaction (0), a vibration around baseline (1), or a true pressure increase (2).

In case the sensor showed calibration deviations at rest (≠0 mmHg), the MeanAct values, Max and AUC were corrected by the pressure (average over 1 s) or AUC measured during rest.

### Electrostimulation

**Figure 1:**
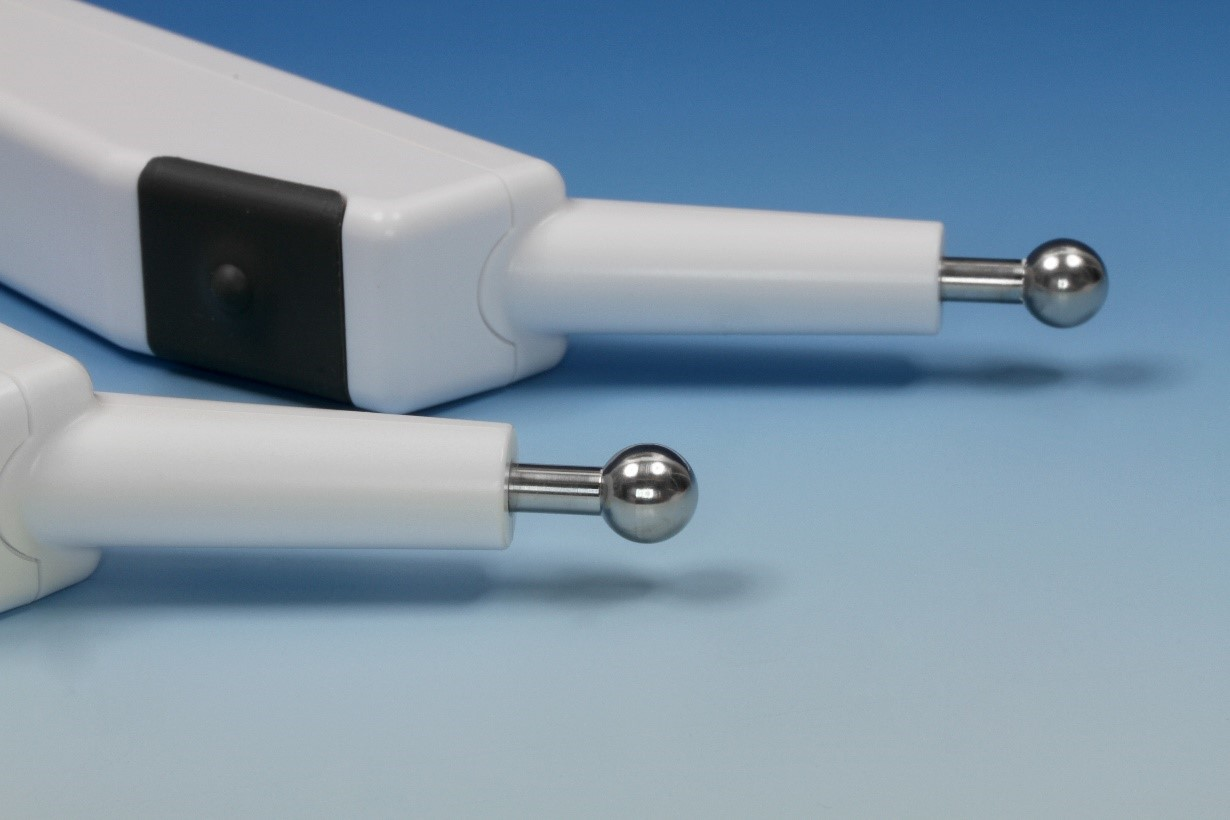
Hand held spot electrodes for electrical stimulation to the velum

**Figure 2:**
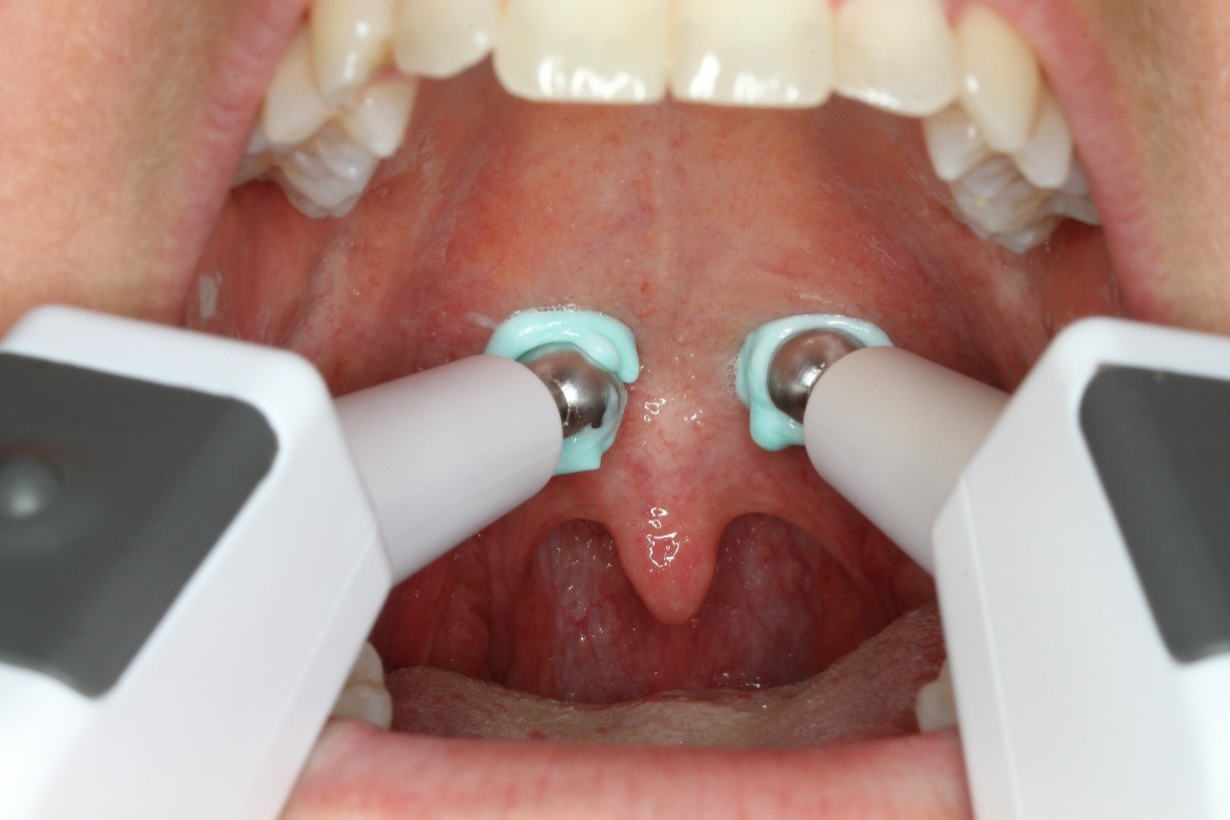
Electrode placement on either side of the midline of the velum through the open mouth

Two handheld electrodes (Figure 1 (Fig. 1)) were placed on either side of the midline of the velum (Figure 2 (Fig. 2)) through the open mouth and held in position. An electric current at medium frequency (KOTS) with a basal frequency of 2,500 Hz and a modulation frequency of 60 Hz was used. Pulse shape was rectangular (including a small ramp) and pulse time 10 ms (device: Physiodyn Expert, Physiomed Elektromedizin AG, Schnaittach, Germany). The start, end and duration of the electric current were determined by manual hand switch and coordinated to the phonation task when necessary. Customary toothpaste (Blend-a-med complete, Schwalbach, Germany) was used as a contact gel.

### Statistics

The intra-individual averages of 10 (individual) realizations of each parameter (per protocol) were initially calculated. Mean values and standard deviations were calculated for all parameters across all participants. A statistical comparison of mean values of isolated phonation (protocol 1) versus the combination of phonation and NMES (protocol 3) was carried out using the Wilcoxon signed rank test. A p-value <5% (p<0.05) was considered significant. The ordinally scaled parameters differentiating the type of velum activity were analyzed using a cross table. The statistical analysis of all collected data was performed using SPSS (Version 24.0; IBM Corp, Armonk, NY, USA).

## Results

Eleven healthy subjects, 5 male and 6 female, with an age range from 21–57 years and a mean age of 33 (median: 32) years were analyzed. 

Mean values (see Table 1 (Tab. 1)) for the phonation associated time period of contraction for protocol 1 (isolated phonation) measured 17.15 SD ±20.69 mmHg on average with mean maximum values of 37.86 SD ±49.17 mmHg. AUC measured 17.06 SD ±20.70 mmHg.s on average. For protocol 3 (phonation + electrostimulation) the period of contraction measured 34.59±25.75 mmHg on average with mean maximum values of 87.24±59.53 mmHg. AUC measured 33.76±23.81 mmHg.s on average. Protocol 2, representing the isolated stimulation, did only produce velum reactions on 32 of the 110 occasions. These presented MeanAct values of 13.58±12.40 mmHg, maximum values of 56.14±53.14 mmHg and AUC values of 13.84±12.78 mmHg.s on average.

Values did not show normal distribution. Statistical analysis comparing the pressures in the velopharyngeal region during isolated phonation (protocol 1) and combined phonation with NMES (protocol 3) showed more positive ranks than negative ones for mean values during the contraction period (8 vs. 3), maximum values (8 vs. 3) and also the AUC (8 vs. 3) during the Wilcoxon signed rank test. Higher values were associated with protocol 3 each time (see Table 2 (Tab. 2)). This difference reached statistical significance (p=0.026) only for maximum values (see Table 2 (Tab. 2)).

The two parameters determining and classifying velum reactions, as ordinal parameters, were analyzed using a crossed table (see Table 3 (Tab. 3)). Protocol 1 and 3 presented with 100% and 96% of velum reactions respectively, under protocol 2 29% of NMES-applications resulted in a manometrically visible reaction. These 29% resulted from 7 out of the 11 subjects investigated and did not always affect all of the 10 electrical stimulation (ES) applications within a subject. As for protocol 1 68% and protocol 3 96% of visible velum reactions were actual pressure increases (as opposed to vibrations across the baseline: 31.8% (protocol 1) and 2.8% (protocol 3)). For protocol 2 26.4% were classified as pressure increases and 1.8% as vibrations, whereas 70.9% represented with no reaction. 

## Discussion

This study aimed to investigate VP pressures during phonation with and without NMES in order to determine whether NMES influences the closing pressures of the VP. The tested parameters did reveal differences in maximum and average pressures between isolated phonation (protocol 1) and phonation combined with NMES (protocol 3). Average Max values during combined phonation and NMES were significantly higher than during isolated phonation of the sound /a/, increasing by more than 200%. In addition, average values over the contraction period were almost twice as high. As mean values for MeanAct, Max and AUC all measured almost or even more than twice as high (200%) for protocol 3 compared to protocol 1, the results show clearly that the application of NMES, as used here, is able to increase VP pressures during phonation. Higher AUC values in association with protocol 3 imply more pressure over time, indicating again that the VPC was more forceful when NMES was applied. An isolated application of NMES (protocol 2) was not able to show pressure increases to the same extent. Nevertheless, an overall increase in pressures of around 14 mmHg for average MeanAct and 56 mmHg for average Max values compared to pressures at rest (0 mmHg) could be detected. It is known that pre-tensed muscles represent with a lower stimulus threshold than muscles at rest [23], [24]. Quite plausibly, the activity support provided by the concurrent NMES was more effective in the “working” muscle than in the relaxed muscle, leading to higher pressures for NMES in combination with phonation as opposed to isolated phonation as well as the effect seen with isolated stimulation. It is quite remarkable, nonetheless, that even though the isolated electrostimulation was only able to activate the muscle in some cases, whenever it was able to activate muscle action the pressure levels were comparable to pressures during the isolated phonation – and with that to the physiologic status during phonation (see Table 1 (Tab. 1)). The data shows that the velopharyngeal closure force can be modified by NMES and that a therapeutical effect may be more effective when targeting and supporting muscle activity rather than stimulation at rest.

Furthermore, rhinophonia aperta is often not only the result of weakened muscle strength, but may result from missing muscle control. NMES might also be able to support this “communication” of the muscle nerve entity by increasing the stimulus given.

The effect the combination of the muscle task (velopharyngeal closure) with NMES has on the velopharynx pressures measured here is rather large. As it can be presumed that higher pressures in the velopharyngeal region coincide with a “better” or more forceful closure during phonation, these results seem very promising especially in relation with therapeutical procedures for velopharyngeal insufficiencies. If a long term increase in muscle strengthening can be achieved this way, however, and if it truly influences the acoustic measures associated with rhinophonia needs to be investigated further. 

Even though pressure differences between average values for protocol 1 and protocol 3 are very similar for MeanAct, Max and AUC values (±200%), a statistical significance only resulted for the Max values. As reported previously for pharyngeal HRM data [4], [20], [21], [25], the standard deviations of the presented data are very high. These large standard deviations might prevent the differences from showing statistical significance even when differences are large and might explain the lack of statistical significance.

The phonation volume was measured for each vocalization. It was noticeable, however, that subjects tended to vocalize at a higher volume during the application of NMES than without, probably due to the strong and “sudden” stimulus. This, however, was checked during the examination via sound level meter, and feedback was given to the individual to adapt their volume. Vocalizations completely outside the accepted volume range from 60 to 70 dB were repeated and not included. An influence of a higher volume associated with utterances in combination with NMES was, therewith, eliminated.

Even though a healthy muscle does generally respond to stimulation above motor threshold by contraction [26], not all the applications of NMES in protocol 2 were able to create a manometrically measurable reaction. Manometrically measurable reactions during the application of isolated ES were seen in 7 out of the 11 subjects. The reactions were not visible across the 10 repetitions for all of these subjects, but generally ranged from 1 to 5 out of the 10 repetitions. One subject, however, presented with 10 clear reactions during protocol 2. This subject seemed to show a relatively strong closure of the velopharynx in general as well as a strong reaction to NMES, as even the production of the sound /a/ was sometimes hindered by an audible blockade of the airflow during combined phonation and ES (protocol 3). Quite possibly in this subject not only the velum but also different structures, such as the M. constrictor pharyngis, were reached by the NMES and formed a constriction or blockage in the pharynx. It may have been a low representation of the gagging reflex every time the current flowed. 

Several other reasons can be put forward with regards to the different amount of manometrically visible reactions in relation with isolated NMES. On the one hand, with some of the subjects stimulation above motor threshold was not possible, as the personal maximum tolerable intensity was reached before a visible muscle twitch was achieved. In these cases the stimulation was carried out just under this personal threshold rather than the desired motor threshold. On the other hand, individual (anatomic) conditions, such as i.e. the individual amount of saliva or different locations of muscle trigger points for velum elevation across the subjects, might be responsible for the inconsistent results in relation with isolated NMES. Nevertheless, even though not all isolated NMES applications were able to create a manometrically visible response, whenever successful, they were able to create pressure increases similar to the increase associated with isolated phonation. 

Active NMES applications were able to enhance VPC pressures during phonation. In healthy subjects, as investigated in this study, this pressure enhancement sometimes interfered with phonation and therefore seemed to be too strong. The phonation of subjects with surgically repaired cleft palate (UCLP), however, could probably benefit from (active) NMES. 

A previous study was able to compare velopharyngeal pressures of healthy individuals and subjects with surgically repaired unilateral cleft palate (UCLP) during the articulation of different sounds and found pressure values of subjects with UCLP to be lower [5]. In a next step it is necessary to investigate, if NMES is able to increase phonation associated pressures of the VP in patients with velopharyngeal impairments, too. This would be particularly interesting with regard to a possible therapeutic effect, which might be achieved in the muscles of the velopharynx, enabling a stronger and, with regards to phonation, a more effective closure.

## Conclusion

As the data shows, NMES does change VP closing pressures during phonation and enhances average pressures of the VPC during phonation by as much as 200% in healthy individuals. It was not possible to measure a consistent muscle contraction in relation with isolated NMES, even though a pressure increase similar to the increase associated with isolated phonation could be shown. This study shows that task supportive NMES is more effective than isolated stimulation and that NMES can change the velopharyngeal closing pattern.

## Notes

### Shared first authorship

Simone Miller and Katharina Peters contributed equally to this work.

### Authors’ contributions

**Simone Miller**

- Conception and design of the work
- Acquisition, analysis, and interpretation of data
- Drafting and revising the work
- Final approval of the version to be published
- Agreement to be accountable for all aspects of the work in ensuring that questions related to the accuracy or integrity of any part of the work are appropriately investigated and resolved

**Katharina Peters**

- Conception and design of the work
- Acquisition, analysis, and interpretation of data
- Drafting and revising the work
- Final approval of the version to be published
- Agreement to be accountable for all aspects of the work in ensuring that questions related to the accuracy or integrity of any part of the work are appropriately investigated and resolved

**Martin Ptok**

- Conception of the work
- Revising the work critically for important intellectual content
- Final approval of the version to be published
- Agreement to be accountable for all aspects of the work in ensuring that questions related to the accuracy or integrity of any part of the work are appropriately investigated and resolved

**Michael Jungheim**

- Conception and design of the work
- Interpretation of data
- Revising the work critically for important intellectual content
- Final approval of the version to be published
- Agreement to be accountable for all aspects of the work in ensuring that questions related to the accuracy or integrity of any part of the work are appropriately investigated and resolved

### Authors’ ORCIDs

- Dr. rer. biol. hum. Simone Miller: 0000-0002-5289-1795
- Katharina Peters, M.Sc.: 0000-0002-3803-8376
- Prof. Dr. med. Dr. med. h.c. Martin Ptok: 0000-0001-6296-1323
- Prof. Dr. med. Michael Jungheim: 0000-0003-4823-3746

### Ethical approval

The study was performed in accordance with the Declaration of Helsinki, Good Clinical Practices, and applicable regulatory requirements and was approved by the ethics committee of the Hannover Medical School (#8611_BO_S_2019). 

### Consent to participate

Written informed consent was obtained from all participants before undergoing any study-related procedures and participants were not financially remunerated.

### Funding

As part of a third party funded project, this work was funded by the AiF Project GmbH of the BMWi (Germany’s Federal Ministry for Economic Affairs and Climate Action), ZF4598701AJ8.

### Competing interests

The authors declare that they have no competing interests.

## Figures and Tables

**Table 1:**
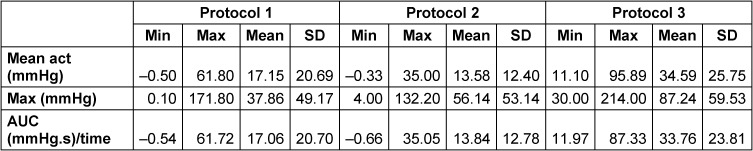
Mean values of all parameters across the three protocols

**Table 2:**
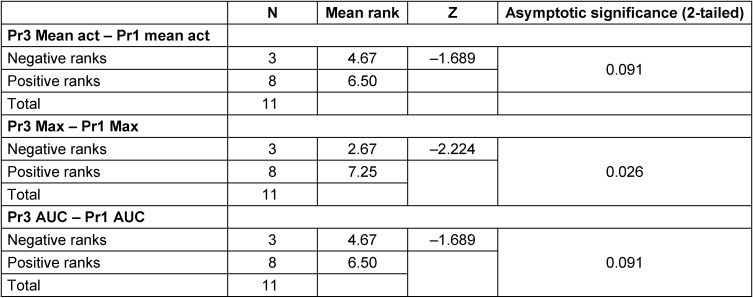
Wilcoxon signed rank test comparing protocol 1 and 3

**Table 3:**
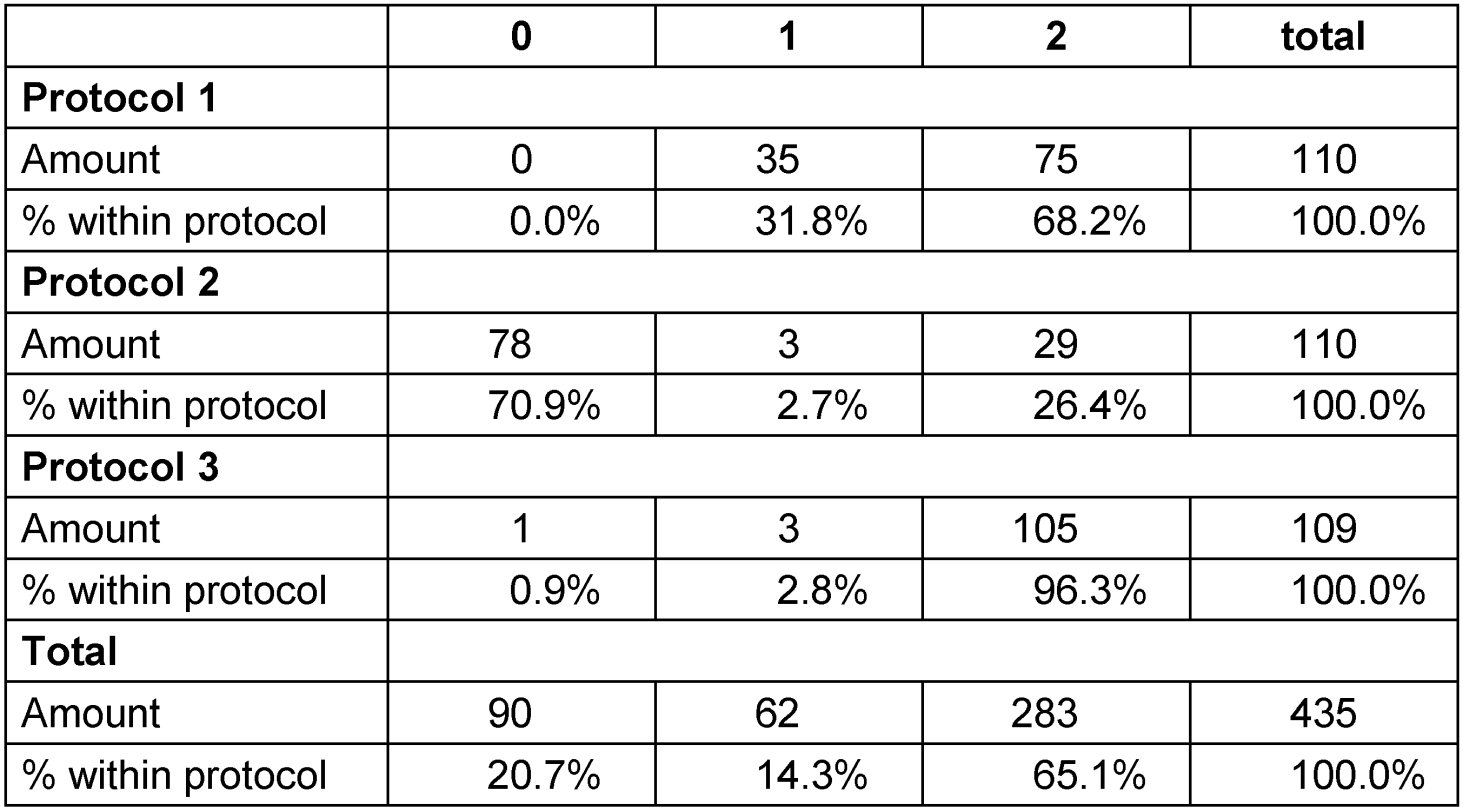
Cross table for the parameter: type of velum activity (0=n/a, 1=vibration, 2=pressure increase)
